# Flow Cytometric Lymphocyte Subset Enumeration for Lymphopenia Grading and C‐Reactive Protein‐Defined Inflammation Discrimination

**DOI:** 10.1002/jcla.70375

**Published:** 2026-10-15

**Authors:** Shicheng Li, Tingting Fu, Yang Yang, Yuwei Pu, Wenying Yan, Junjun Zhang

**Affiliations:** ^1^ School of Computing Science and Artificial Intelligence Suzhou City University Suzhou China; ^2^ Suzhou Key Lab of Multi‐Modal Data Fusion and Intelligent Healthcare Suzhou City University Suzhou China; ^3^ Department of Radiology The Fourth Affiliated Hospital of Soochow University, Suzhou Dushu Lake Hospital Suzhou China; ^4^ Department of General Surgery The Second Affiliated Hospital of Soochow University Suzhou China; ^5^ Key Laboratory of Symbolic Computation and Knowledge Engineering of Ministry of Education Jilin University Changchun China; ^6^ PRaG Center, Center for Cancer Diagnosis and Treatment The Second Affiliated Hospital of Soochow University Suzhou China

**Keywords:** B lymphocytes, C‐reactive protein, flow cytometry, lymphopenia, T lymphocytes

## Abstract

**Background:**

To determine whether flow cytometric lymphocyte subset enumeration could reproduce conventional absolute lymphocyte count (ALC)‐based lymphopenia grading and add information for discriminating C‐reactive protein (CRP)‐defined inflammation.

**Methods:**

We retrospectively analyzed 2896 hospitalized adults with concurrent complete blood count and flow cytometric lymphocyte subset analysis (T, CD4+ T, CD8+ T, B, and natural killer cells). CRP‐defined inflammation was defined as high‐sensitivity CRP ≥ 10 mg/L. The 974 patients with measured CRP constituted the prespecified primary discrimination cohort. Spearman correlations, receiver operating characteristic analyses with Youden‐derived cutoffs, quadratic‐weighted kappa, five‐fold internal validation, and multivariable logistic regression were used.

**Results:**

T‐cell absolute count correlated most strongly with ALC (rho = 0.886), largely reflecting its dominant contribution to the total lymphocyte pool. Internally optimized T‐cell cutoffs of 700, 573, and 356 cells/μL showed substantial agreement with conventional grading (weighted kappa = 0.792). For CRP‐defined inflammation (20.9%), conventional and T‐cell grades showed only modest discrimination (areas under the curve, 0.611–0.622). B‐cell count had the numerically highest single‐marker area under the curve (0.667), although all single‐marker values remained below clinically actionable thresholds. Parsimonious multivariable models incorporating B‐cell count yielded modest cross‐validated areas under the curve (0.628–0.642), whereas a full five‐subset model exhibited severe collinearity and unstable coefficients.

**Conclusion:**

T‐cell count serves as an internally optimized surrogate for conventional lymphopenia grading rather than an independent classifier. For CRP‐defined inflammation, lymphocyte subset models offer limited cross‐sectional discrimination, with B‐cell count providing the most reproducible incremental signal.

## Graphical abstract

This study refines conventional lymphopenia grading by establishing optimized T‐cell cutoffs via flow cytometric lymphocyte subset profiling. Additionally, by evaluating individual subset performance (CD4^+^, CD8^+^, B, and NK cells), the framework integrates these counts to construct predictive models for infection‐associated inflammation.

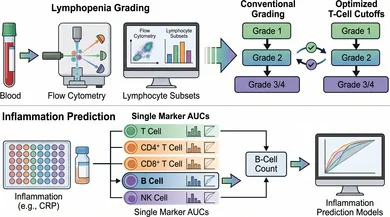

## 1 Introduction

Peripheral blood lymphopenia is a frequent laboratory abnormality in hospitalized patients with infection, malignancy, autoimmune disease, transplantation, chemotherapy, radiotherapy, and immunosuppressive therapy. The Common Terminology Criteria for Adverse Events grading system classifies lymphopenia by absolute lymphocyte count (ALC), providing a simple and standardized severity framework [1]. Population‐based and hospitalized‐cohort evidence has linked lymphopenia with infection and adverse outcomes, although the strength and specificity of this association vary by clinical context [2, 3].

Although this grade is convenient, it compresses several biologically different immune compartments into a single number. The total lymphocyte count does not distinguish between T‐cell‐predominant depletion, B‐cell depletion, NK‐cell depletion, or mixed patterns [4]. This distinction matters because these subsets do not contribute interchangeably to host defense: CD4+ T cells coordinate cellular and humoral responses, CD8+ T cells mediate cytotoxic antiviral and antitumor activity, B cells support antibody‐mediated immunity, and NK cells participate in early innate cytotoxic responses [5, 6, 7, 8].

Flow cytometric lymphocyte subset enumeration is therefore attractive as a more granular laboratory description of immune status. In clinical practice, however, this additional granularity is not automatically equivalent to better clinical discrimination. A subset that represents most circulating lymphocytes may closely mirror ALC simply because of arithmetic composition, whereas a smaller subset may carry complementary biological information that is not visible from ALC alone. Distinguishing these two possibilities is important before proposing a flow‐based grading scheme.

A further challenge is the outcome framework. Infection status is often difficult to define retrospectively, and inflammatory biomarkers such as C‐reactive protein (CRP) are commonly measured at clinician discretion rather than uniformly across all patients. CRP is a well‐established acute‐phase marker of inflammation, but it is not specific for infection and can be elevated in diverse noninfectious inflammatory states [9, 10, 11]. Selective CRP ordering also creates a missing‐data problem: patients without CRP testing cannot safely be treated as uninfected, because the decision to order CRP may itself reflect clinical suspicion of infection or inflammation [12]. Analyses that use CRP‐defined outcomes therefore need to separate patients with measured CRP from those with unmeasured CRP. CRP is synthesized predominantly in response to cytokine‐driven innate immune activation, whereas ALC and most lymphocyte subsets describe circulating adaptive immune compartments (with NK cells contributing innate cytotoxicity); accordingly, neither measurement is expected to serve as a universal surrogate for the other. Systemic inflammation may nevertheless be accompanied by context‐dependent lymphocyte redistribution, apoptosis, marrow suppression, treatment‐related depletion, or disease‐specific subset loss. We therefore evaluated whether subset counts provided complementary cross‐sectional information for CRP‐defined inflammation, not whether flow cytometry could replace CRP.

This study addressed three linked questions: first, which flow cytometric marker most closely tracks conventional ALC‐based lymphopenia grading; second, what internally optimized T‐cell cutoffs best reproduce that grading; and third, whether subset information is associated with CRP‐defined inflammation among patients in whom CRP was actually measured. Because T cells make up the largest fraction of circulating lymphocytes, the first two questions were treated as surrogate‐mapping analyses rather than independent prediction tasks, while the third question focused on cross‐sectional discrimination rather than causal inference.

## 2 Materials and Methods

### 2.1 Study Population

This retrospective single‐center study included adult hospitalized patients (≥ 18 years) who underwent complete blood count and multicolor flow cytometric lymphocyte subset analysis during the same hospitalization. Patients were eligible if absolute lymphocyte count and absolute counts of the major lymphocyte subsets were available, allowing assignment of conventional lymphocyte grades and evaluation of flow cytometric subset counts. High‐sensitivity C‐reactive protein (hs‐CRP) was recorded when available and was used for the complete‐case CRP‐defined inflammation analysis. For patients with multiple eligible hospitalizations during the study period, only the first eligible hospitalization was included. Patients were excluded if they were younger than 18 years, had missing complete blood count data, lacked flow cytometric lymphocyte subset results, or had incomplete key laboratory data required for lymphocyte grading and subset‐based analyses. Patients without measured hs‐CRP were not excluded from analyses of lymphocyte grading and subset correlations, but they were excluded from the primary CRP‐defined inflammation analysis. The study was conducted under institutional review board approval with informed consent waived for retrospective anonymized analysis, and the reporting framework was aligned with principles for observational studies [13]. Ethical approval for this study (JD‐HG‐2026‐044) was provided by the Ethical Committee of The Second Affiliated Hospital of Soochow University. Written informed consent was waived.

### 2.2 Laboratory Measurements

Complete blood counts were measured using a Sysmex XN‐3000 hematology analyzer (Sysmex Corporation, Kobe, Japan). Flow cytometry was performed using a BD LSR II Special Order flow cytometer configured with 11 fluorescence channels (BD Biosciences, San Jose, CA, USA). Archived laboratory documentation identified 8‐color and 10‐color antibody programs. For the cell populations analyzed in this study, the recorded lineage definitions were T cells (CD3^+^), CD4^+^ T cells (CD3^+^CD4^+^CD8^−^), CD8^+^ T cells (CD3^+^CD8^+^CD4^−^), B cells (CD19^+^CD20^+^), and NK cells (CD3^−^CD56^+^). Absolute subset counts were obtained by a dual‐platform calculation combining lymphocyte percentages determined by flow cytometry with absolute lymphocyte counts retrieved from the automated complete blood count [14, 15, 16].

Sample handling, staining, acquisition, gating, internal quality control, and external quality‐assurance procedures were conducted at the Department of Laboratory Medicine of the Second Affiliated Hospital of Soochow University following standard institutional operating procedures. Briefly, 2 mL of peripheral venous blood was collected into EDTA‐K2 vacuum anticoagulant tubes between 8:00 AM and 11:00 AM and processed immediately to maintain cell viability. Whole blood (50 μL) was stained using a direct immunolabeling method for 15–20 min at room temperature in the dark. Erythrocytes were subsequently lysed using the OptiLyse C lysing solution (Beckman Coulter, Brea, CA, USA) for 10 min under identical environmental conditions. Data acquisition and review were independently verified by two experienced laboratory technicians. Flow cytometry data were analyzed using FlowJo v8 software (FlowJo LLC, Ashland, OR, USA). Specimens for CBC, flow cytometry, and hs‐CRP were drawn simultaneously via a single venipuncture. According to institutional laboratory protocols, all samples were processed within standardized time windows to ensure sample integrity: whole‐blood flow cytometric staining and CBC analysis were completed within 3 h of collection, and serum hs‐CRP was separated within 3 h and analyzed within 3 h. High‐sensitivity CRP was measured by latex particle‐enhanced immunoturbidimetry on a cobas c 503 automated chemistry module using the Tina‐quant Cardiac high sensitivity CRP III assay (catalog no. 09188231190; Roche Diagnostics GmbH, Mannheim, Germany).

### 2.3 Definitions and Outcomes

Conventional lymphocyte grade was assigned as normal (ALC ≥ 1.1 × 10^9^/L), Grade 1 (0.8 to < 1.1 × 10^9^/L), Grade 2 (0.5 to < 0.8 × 10^9^/L), Grade 3 (0.2 to < 0.5 × 10^9^/L), and Grade 4 (< 0.2 × 10^9^/L). Because Grade 4 was rare, Grades 3 and 4 were combined. CRP‐defined inflammation was defined as CRP ≥ 10 mg/L. Since CRP was measured in only 974 of 2896 patients, the CRP‐measured complete‐case cohort was used for the primary discrimination analysis. Treating unmeasured CRP as negative was retained only as a sensitivity analysis because CRP testing was likely missing not at random. The 10 mg/L threshold was prespecified because it was the upper limit of the assay reference interval and was intended to distinguish overt inflammatory responses from low‐grade inflammation. This choice is also consistent with the CDC/AHA scientific statement recommending that hs‐CRP values ≥ 10 mg/L prompt evaluation for an acute infectious or inflammatory source and repeat testing after clinical stability [17].

### 2.4 Statistical Analysis

Continuous variables are summarized as median (IQR), and group comparisons used rank‐based tests. Categorical variables were compared using chi‐square tests. Spearman rho quantified associations between subset markers and both ALC and conventional grade; the count‐grade correlation was interpreted cautiously because grade is derived directly from ALC. Areas under the receiver operating characteristic curve and Youden cutoffs were calculated for conventional grade mapping and CRP‐defined inflammation [18, 19]. Bootstrap 95% confidence intervals were used for areas under the receiver operating characteristic curve. Pairwise AUC comparisons used a bootstrap implementation for correlated ROC curves based on the rationale of comparing correlated areas under the receiver operating characteristic curve [20]. Five‐fold internal validation was used to evaluate cutoff‐derived T‐cell grade agreement and logistic‐model discrimination [21]. Logistic models used standardized cell counts and were reported with attention to model‐performance reporting principles [22]; variance inflation factors (VIFs) assessed collinearity. Analyses were performed using Python version 3.9.

## 3 Results

### 3.1 Baseline Characteristics and CRP Missingness

Baseline characteristics by conventional lymphocyte grade are summarized in Table 1. The full cohort included 2896 patients, comprising 1370 with normal ALC, 734 with Grade 1, 590 with Grade 2, and 202 with Grade 3/4 lymphopenia. CRP was measured in 974 patients (33.6%), with similar measurement frequencies across conventional grades (30.2%–34.5%, *p* = 0.676); therefore, CRP‐based analyses were restricted to the measured subgroup rather than treating missing CRP values as negative. Lymphopenia severity was accompanied by a stepwise decline in ALC from 1.40 × 10^9^/L in the normal group to 0.40 × 10^9^/L in Grade 3/4, and absolute lymphocyte subset counts decreased in parallel: median T‐cell count declined from 945.0 to 258.0 cells/μL, CD4+ T cells from 535.0 to 122.5 cells/μL, CD8+ T cells from 401.5 to 107.0 cells/μL, B cells from 120.0 to 15.0 cells/μL, and NK cells from 290.0 to 60.5 cells/μL. In contrast, subset percentages changed less consistently, with no significant difference in CD4+ T‐cell percentage and only borderline variation in NK‐cell percentage. Among patients with measured CRP, the proportion with CRP ≥ 10 mg/L increased from 15.7% in the normal group to 47.5% in Grade 3/4, supporting a graded but nonspecific association between lymphopenia severity and CRP‐defined inflammation.

**TABLE 1:** Baseline characteristics by conventional lymphocyte grade.

Variable	Overall	Normal (≥ 1.1)	Grade 1 (0.8–< 1.1)	Grade 2 (0.5–< 0.8)	Grade 3/4 (< 0.5)	*p*
*N*	2896	1370	734	590	202	
CRP measured, *n* (%)	974 (33.6%)	472 (34.5%)	244 (33.2%)	197 (33.4%)	61 (30.2%)	0.676
WBC (×10^9^/L)	5.10 (4.00–6.60)	5.70 (4.60–7.20)	4.80 (3.90–6.10)	4.40 (3.40–6.00)	4.00 (2.90–5.95)	< 0.001
Lymphocytes (×10^9^/L)	1.00 (0.70–1.40)	1.40 (1.20–1.80)	0.90 (0.80–1.00)	0.60 (0.50–0.70)	0.40 (0.30–0.40)	< 0.001
Neutrophils (×10^9^/L)	3.40 (2.50–4.80)	3.50 (2.60–4.80)	3.40 (2.50–4.60)	3.20 (2.30–4.80)	3.20 (2.20–4.97)	0.021
Monocytes (×10^9^/L)	0.40 (0.30–0.60)	0.50 (0.33–0.60)	0.40 (0.30–0.50)	0.40 (0.29–0.50)	0.34 (0.28–0.50)	< 0.001
Eosinophils (×10^9^/L)	0.09 (0.04–0.16)	0.10 (0.05–0.19)	0.08 (0.04–0.16)	0.07 (0.03–0.14)	0.05 (0.02–0.10)	< 0.001
Basophils (×10^9^/L)	0.02 (0.01–0.04)	0.03 (0.02–0.04)	0.02 (0.01–0.03)	0.02 (0.01–0.03)	0.01 (0.01–0.02)	< 0.001
Hemoglobin (g/L)	119.0 (105.0–131.2)	124.0 (112.0–136.0)	118.0 (105.0–130.0)	112.0 (98.0–124.0)	105.0 (88.0–118.8)	< 0.001
Platelets (×10^9^/L)	187.0 (136.0–240.0)	204.0 (154.0–259.0)	181.5 (133.0–238.0)	164.0 (116.0–216.0)	147.5 (102.0–195.8)	< 0.001
T lymphocytes (cells/μL)	672.0 (462.0–934.2)	945.0 (780.0–1192.8)	605.5 (510.2–700.0)	423.0 (354.0–498.0)	258.0 (176.0–310.0)	< 0.001
CD4+ T cells (cells/μL)	356.5 (236.0–533.0)	535.0 (409.0–680.0)	318.0 (250.0–398.8)	223.0 (170.0–275.8)	122.5 (85.2–171.5)	< 0.001
CD8+ T cells (cells/μL)	292.0 (193.0–416.0)	401.5 (303.0–545.0)	273.0 (203.0–345.0)	190.5 (148.0–248.8)	107.0 (76.2–161.8)	< 0.001
B lymphocytes (cells/μL)	75.0 (33.0–139.0)	120.0 (72.0–196.8)	63.0 (33.0–111.8)	37.0 (16.2–68.0)	15.0 (8.0–23.0)	< 0.001
NK cells (cells/μL)	193.0 (112.0–310.0)	290.0 (183.2–430.0)	187.0 (123.0–255.8)	114.0 (77.0–167.8)	60.5 (39.0–94.5)	< 0.001
T lymphocytes (%)	68.9 (60.2–77.3)	67.9 (59.5–75.7)	68.3 (59.6–76.8)	70.8 (61.4–79.0)	73.8 (64.1–80.4)	< 0.001
CD4+ T cells (%)	37.1 (29.5–44.5)	37.5 (30.2–44.6)	36.5 (29.0–44.3)	37.2 (29.4–44.2)	37.0 (27.6–45.4)	0.424
CD8+ T cells (%)	29.7 (22.9–37.2)	28.4 (21.9–35.9)	30.2 (23.4–37.4)	31.4 (24.7–39.1)	32.3 (25.8–42.4)	< 0.001
B lymphocytes (%)	7.3 (4.0–11.9)	8.4 (4.9–12.7)	7.2 (3.8–12.3)	5.9 (2.8–10.7)	4.4 (2.3–6.9)	< 0.001
NK cells (%)	19.9 (13.3–28.0)	20.1 (13.3–28.5)	21.0 (14.0–28.1)	19.1 (12.8–27.1)	18.8 (13.0–26.0)	0.052
CRP among measured (mg/L)	5.3 (5.1–7.1)	5.3 (5.0–5.8)	5.3 (5.1–7.0)	5.3 (5.1–12.4)	6.3 (5.3–48.5)	< 0.001
CRP ≥ 10 mg/L among measured, *n* (%)	204 (20.9%)	74 (15.7%)	47 (19.3%)	54 (27.4%)	29 (47.5%)	< 0.001

*Note:* Data are median (interquartile range) unless otherwise indicated. CRP summaries and CRP ≥ 10 mg/L rates are restricted to patients with measured CRP.

Abbreviations: ALC, absolute lymphocyte count; CRP, C‐reactive protein; NK, natural killer; WBC, white blood cell.

### 3.2 Subset Markers Tracking Conventional Lymphocyte Grading

Absolute subset counts, especially T‐cell count, strongly tracked conventional ALC and ALC‐derived grade (Figures 1 and S1). T‐cell count had the strongest correlation with ALC (rho = 0.886) and conventional grade (rho = −0.816), followed by CD4+ T cells (rho = 0.818 with ALC; rho = −0.759 with grade) and CD8+ T cells (rho = 0.728 with ALC; rho = −0.658 with grade). NK‐cell and B‐cell absolute counts were also correlated with ALC but less strongly (rho = 0.638 and 0.592, respectively). By contrast, subset percentages showed weak or inconsistent correlations with ALC and grade, indicating that absolute subset counts, rather than relative proportions, were the relevant variables for reproducing conventional lymphocyte grading. These findings support T‐cell count as the closest flow‐cytometric surrogate for total lymphocyte count, while also underscoring that this relationship largely reflects the dominant contribution of T cells to the total lymphocyte pool. Spearman correlations between flow cytometric markers and conventional lymphocyte indices are summarized in Table 2.

**FIGURE 1:**
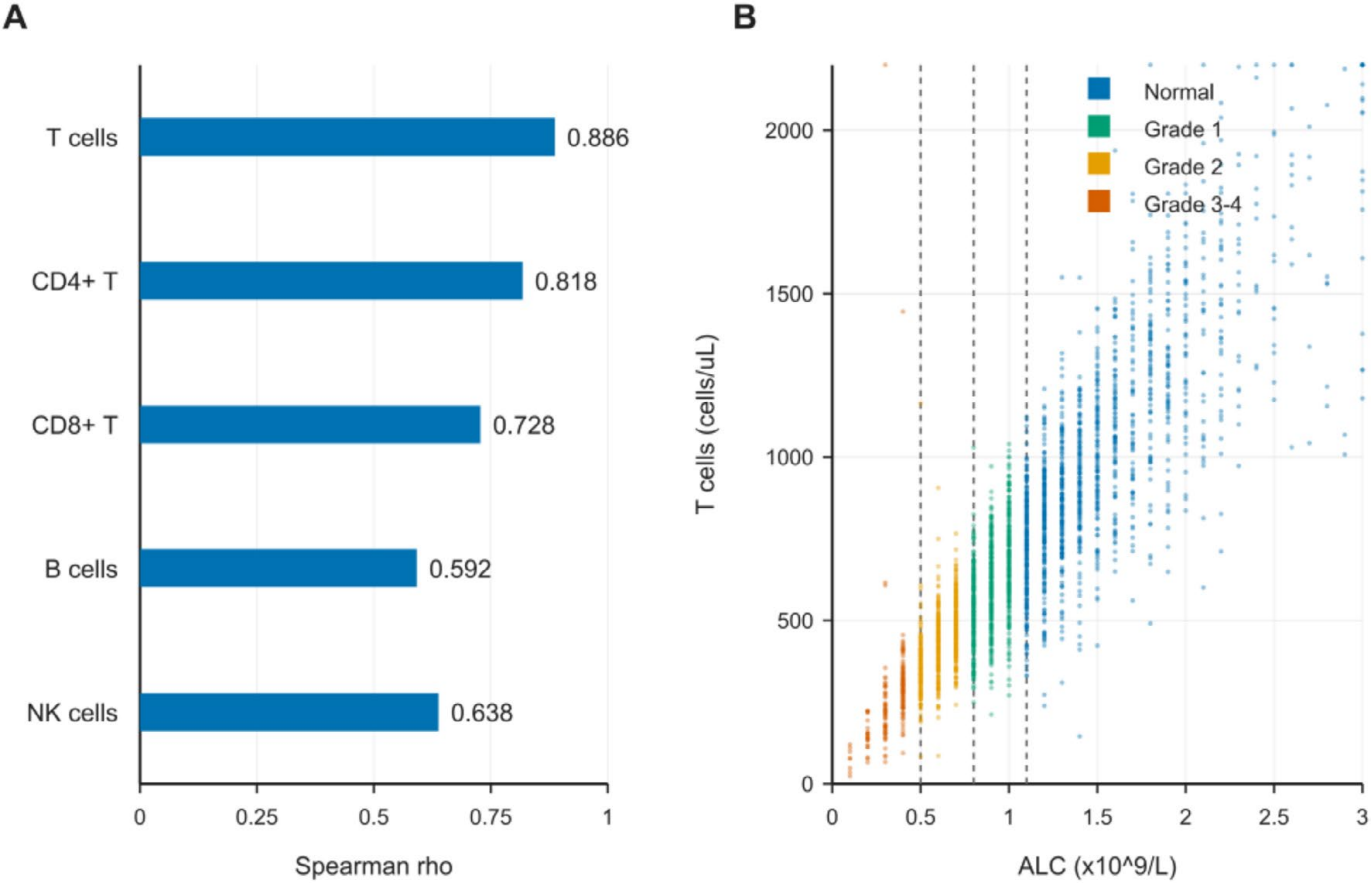
Correlations between flow cytometric subset counts and absolute lymphocyte count. (A) Spearman rho values for each absolute subset count. (B) T‐cell count versus absolute lymphocyte count, colored by conventional grade. Vertical dashed lines indicate conventional absolute lymphocyte count grade thresholds.

**TABLE 2:** Spearman correlations between flow cytometric markers and conventional lymphocyte indices.

Marker	Rho with lymphocyte count (absolute count)	Rho with grade (absolute count)	Rho with lymphocyte count (%)	Rho with grade (%)
T lymphocytes	0.886	−0.816	−0.096	0.111
CD4+ T cells	0.818	−0.759	0.023	−0.025
CD8+ T cells	0.728	−0.658	−0.118	0.134
B lymphocytes	0.592	−0.558	0.191	−0.196
NK cells	0.638	−0.599	0.019	−0.031

Abbreviations: ALC, absolute lymphocyte count; NK, natural killer; rho, Spearman rank correlation coefficient.

### 3.3 Same‐Data ROC Performance for Conventional Grade Thresholds

In same‐data ROC analyses, T‐cell count provided the highest discrimination for each conventional lymphopenia threshold, as illustrated in Figure 2. For identifying any lymphopenia (Grade ≥ 1), T‐cell count achieved an AUC of 0.933, exceeding CD4+ T cells (AUC 0.902), CD8+ T cells (AUC 0.843), NK cells (AUC 0.804), and B cells (AUC 0.788). The same pattern was observed for moderate‐to‐severe lymphopenia (Grade ≥ 2), where T‐cell count had an AUC of 0.942, and for severe lymphopenia (Grade ≥ 3), where the AUC increased to 0.962. The corresponding Youden cutoffs for T‐cell count were 700, 573, and 356 cells/μL, with apparent sensitivities of 0.876, 0.934, and 0.896 and apparent specificities of 0.851, 0.817, and 0.930, respectively. Thus, T‐cell count numerically reproduced ALC‐defined thresholds better than other subsets, but the analysis should be interpreted as an internally optimized mapping exercise rather than independent validation. Detailed same‐data performance for all flow cytometric markers is provided in Supporting Information (Table S1).

**FIGURE 2:**
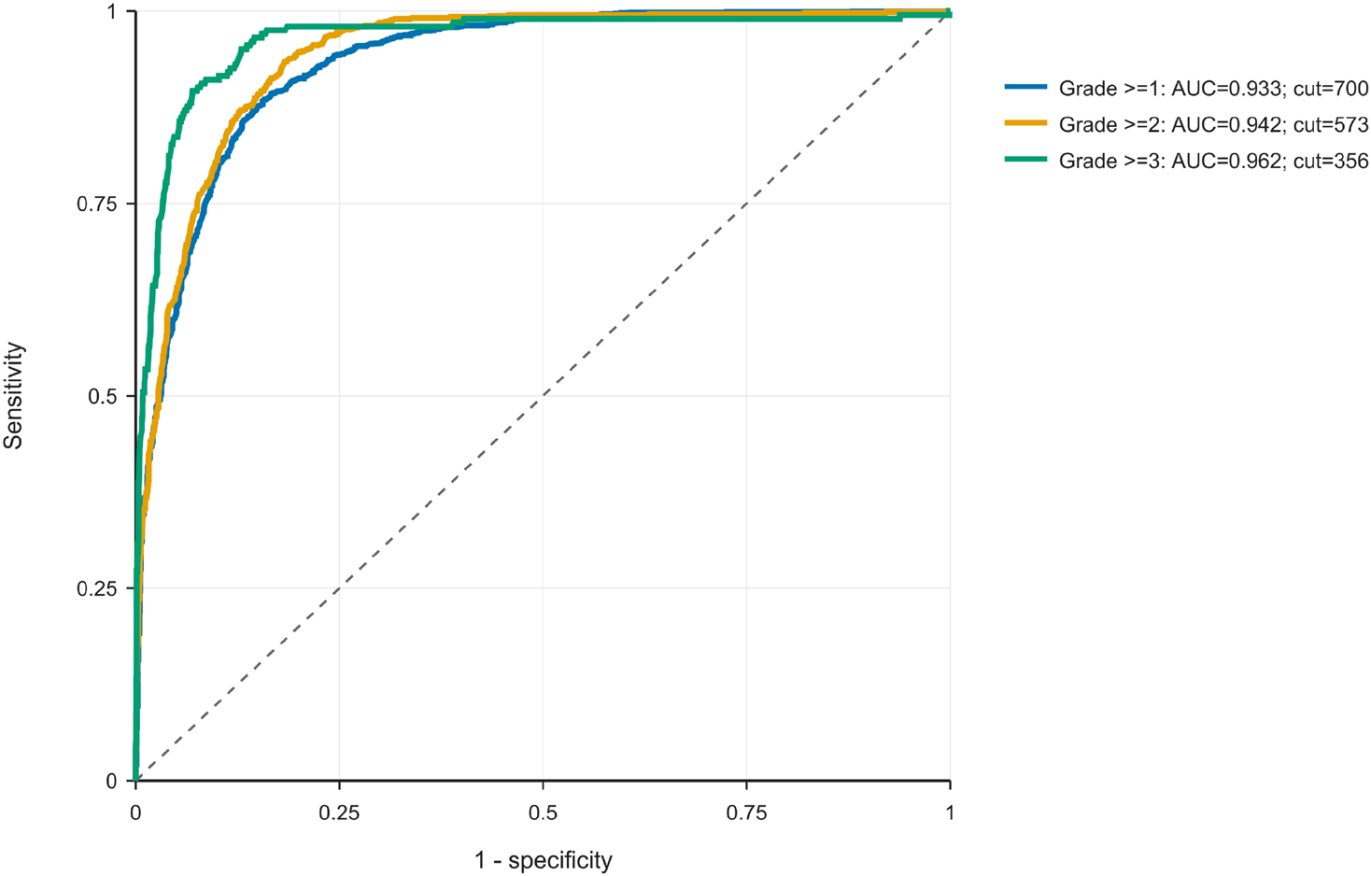
Same‐data receiver operating characteristic curves for T‐cell absolute count identifying conventional lymphopenia thresholds. These curves map a dominant lymphocyte subset onto an absolute lymphocyte count‐derived grade and therefore represent internally optimized surrogate analyses.

### 3.4 Construction and Internal Validation of a T‐Cell‐Based Grade

Using the same‐data Youden thresholds, T‐cell counts of 700, 573, and 356 cells/μL defined a four‐category T‐cell grade. Apparent agreement with conventional grading was substantial (weighted kappa 0.792), and the cross‐classification matrix showed that most observations fell on or near the diagonal: 1166 of 1370 patients with normal conventional grade were also classified as normal by the T‐cell grade, and 179 of 202 conventional Grade 3/4 patients were classified as T‐cell Grade 3/4. Misclassification was concentrated mainly between adjacent middle categories, consistent with overlapping continuous count distributions. Five‐fold internal validation yielded a mean weighted kappa of 0.782, with validated sensitivities of 0.869, 0.930, and 0.896 and specificities of 0.851, 0.817, and 0.912 for the Grade ≥ 1, Grade ≥ 2, and Grade ≥ 3 thresholds, respectively. These results indicate reproducible within‐dataset alignment between T‐cell grade and conventional grade, while still requiring external validation before clinical adoption. T‐cell cutoffs and internally validated agreement with conventional grade are shown in Table 3.

**TABLE 3:** T‐cell cutoffs and internally validated agreement with conventional grade.

Target conventional threshold	Apparent cutoff (cells/μL)	Apparent AUC	Apparent Sens	Apparent Spec	5‐fold validated Sens	5‐fold validated Spec
Grade ≥ 1	700.0	0.933	0.876	0.851	0.869	0.851
Grade ≥ 2	573.0	0.942	0.934	0.817	0.930	0.817
Grade ≥ 3	356.0	0.962	0.896	0.930	0.896	0.912
Weighted kappa vs. conventional grade	700/573 / 356	0.792			5‐fold mean 0.782	Range 0.714–0.811

Abbreviations: AUC, area under the receiver operating characteristic curve; Sens, sensitivity; Spec, specificity.

### 3.5 CRP‐Defined Inflammation

Primary discrimination analyses were restricted to the 974 patients with measured CRP, among whom 204 had CRP ≥ 10 mg/L (Figures 3 and S2). Conventional grade, new T‐cell grade, ALC, and T‐cell count all showed modest discrimination, with areas under the receiver operating characteristic curve clustered between 0.611 and 0.622. B‐cell count had the numerically highest single‐marker AUC (0.667, 95% CI 0.627–0.709), using an optimal cutoff of 39 cells/μL with sensitivity 0.485 and specificity 0.783; however, all single‐marker areas under the receiver operating characteristic curve remained below a level that would support stand‐alone clinical classification. The full‐cohort missing‐as‐negative sensitivity analysis produced uniformly lower areas under the receiver operating characteristic curve, including 0.630 for B cells, 0.603 for CD4+ T cells, 0.595 for T cells, 0.590 for T‐cell grade, and 0.587 for conventional grade, consistent with dilution from outcome misclassification when unmeasured CRP values are assumed to be negative. Overall, the CRP‐associated signal was strongest for B‐cell count but remained limited in absolute discriminative performance. Single‐marker discrimination for C‐reactive protein ≥ 10 mg/L in the measured complete‐case cohort is shown in Table 4.

**FIGURE 3:**
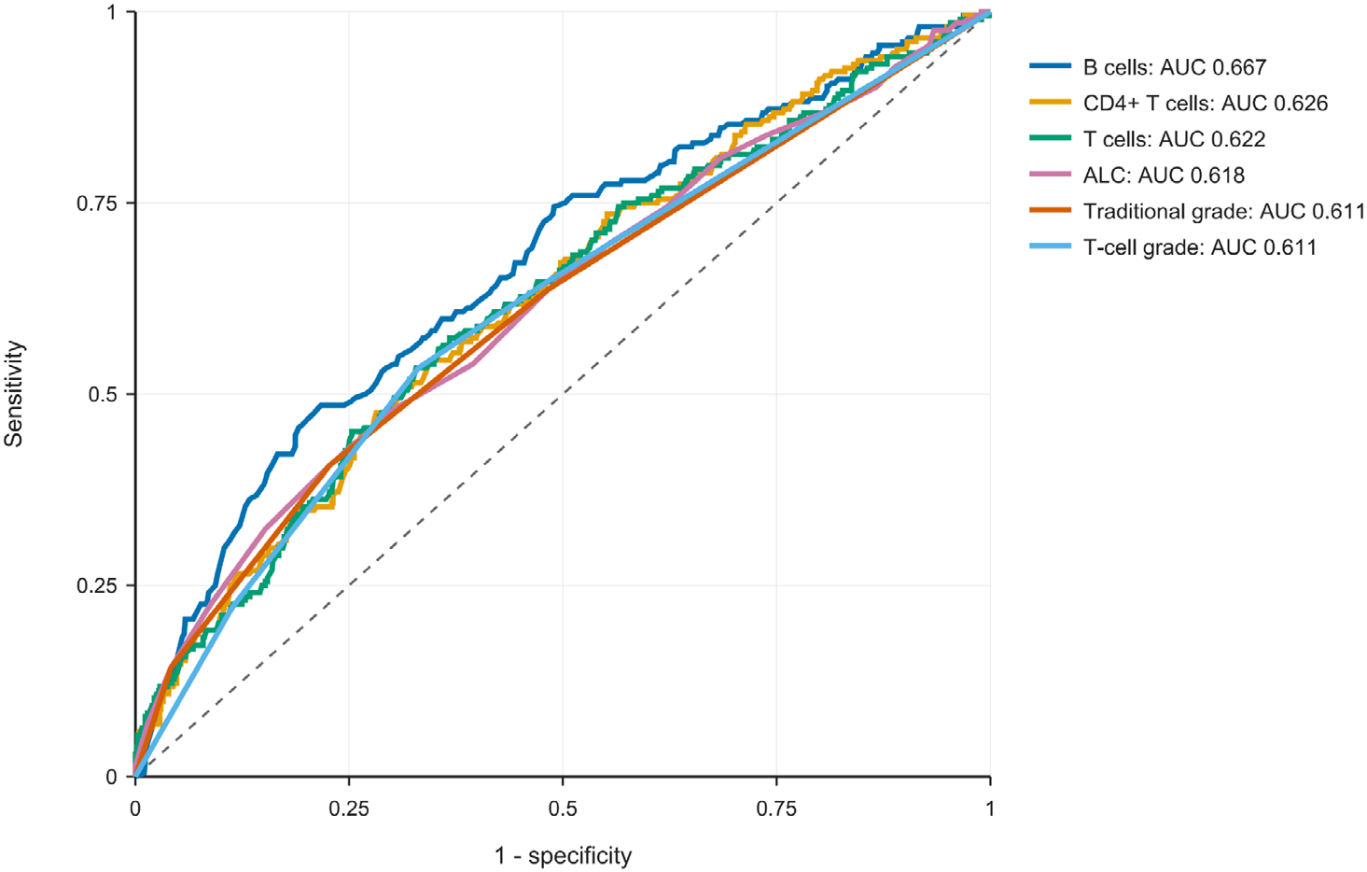
Receiver operating characteristic curves for CRP ≥ 10 mg/L among patients with measured CRP (*n* = 974; events = 204). B‐cell count has the numerically highest area under the curve among the displayed single markers, whereas conventional and T‐cell grades show similarly modest discrimination.

**TABLE 4:** Single‐marker discrimination for CRP ≥ 10 mg/L in the measured complete‐case cohort.

Predictor	AUC	95% CI	Cutoff	Sensitivity	Specificity	Bootstrap *p* vs. B‐cell count	Full‐cohort AUC
Lymphocyte count (continuous, ×10^9^/L)	0.618	0.573–0.662	0.7	0.407	0.773	0.023	0.588
Traditional lymphocyte grade (0–3)	0.611	0.568–0.653	2.0	0.407	0.773	0.009	0.587
T lymphocyte absolute count (cells/μL)	0.622	0.575–0.665	601.0	0.574	0.632	0.043	0.595
CD4+ T cell absolute count (cells/μL)	0.626	0.580–0.666	305.0	0.539	0.658	0.047	0.603
CD8+ T cell absolute count (cells/μL)	0.600	0.555–0.646	251.0	0.520	0.664	0.011	0.564
B lymphocyte absolute count (cells/μL)	0.667	0.627–0.709	39.0	0.485	0.783		0.630
NK cell absolute count (cells/μL)	0.571	0.525–0.616	144.0	0.461	0.681	< 0.001	0.553
New T‐cell based grade (0–3)	0.611	0.568–0.652	2.0	0.534	0.669	0.009	0.590

*Note:* Pairwise *p*‐values versus B‐cell count were estimated by bootstrap comparison of correlated areas under the curve.

Abbreviations: AUC, area under the receiver operating characteristic curve; CI, confidence interval; CRP, C‐reactive protein; NK, natural killer.

### 3.6 Multivariable Models and Collinearity Diagnostics

Multivariable models showed only modest incremental discrimination and highlighted substantial collinearity among redundant T‐cell‐family predictors. Traditional grade alone had an apparent AUC of 0.611 and a five‐fold cross‐validated AUC of 0.590, while the new T‐cell grade alone produced nearly identical values (0.611 and 0.592). Adding B‐cell count to conventional grade increased the apparent AUC to 0.658 and the cross‐validated AUC to 0.628, with both higher grade (aOR 1.315 per grade, *p* = 0.003) and lower B‐cell count (aOR 0.490 per 1 SD increase, *p* < 0.001) independently associated with CRP ≥ 10 mg/L. The nonredundant T + B + NK panel yielded the highest cross‐validated AUC (0.642), but discrimination remained moderate. In the exploratory five‐subset model, VIFs were extremely high for T cells, CD4+ T cells, and CD8+ T cells (763.7, 274.1, and 217.2), explaining the unstable T‐cell‐family coefficients and cautioning against biological interpretation of the NK‐cell direction in that model. These results support the conclusion that B‐cell count adds limited complementary information, whereas replacing conventional grade with a T‐cell grade alone does not improve CRP‐defined discrimination. Logistic models for C‐reactive protein ≥ 10 mg/L in the measured complete‐case cohort are summarized in Table 5.

**TABLE 5:** Logistic models for CRP ≥ 10 mg/L in the measured complete‐case cohort.

Model	Predictors	AUC	5‐fold AUC	Key coefficients
Model 1: traditional grade	Conventional grade	0.611	0.590	Grade: 1.569 (1.342–1.834), *p* < 0.001
Model 2: new T‐cell grade	T‐cell grade	0.611	0.592	T‐cell grade: 1.441 (1.259–1.649), *p* < 0.001
Model 3: five subset panel (exploratory)	T‐cell, CD4+ T‐cell, CD8+ T‐cell, B‐cell, NK‐cell counts	0.671	0.636	T‐cell: 21.028 (0.273–1617.676), *p* = 0.169; CD4+ T‐cell: 0.114 (0.009–1.533), *p* = 0.101; CD8+ T‐cell: 0.194 (0.019–1.974), *p* = 0.166; B‐cell: 0.443 (0.292–0.674), *p* < 0.001; NK‐cell: 1.206 (1.026–1.418), *p* = 0.023
Model 4: traditional grade + B cells	Conventional grade, B‐cell count	0.658	0.628	Grade: 1.315 (1.098–1.575), *p* = 0.003; B‐cell: 0.490 (0.323–0.744), *p* < 0.001
Model 5: nonredundant T + B + NK panel	T‐cell, B‐cell, NK‐cell counts	0.669	0.642	T‐cell: 0.731 (0.584–0.915), *p* = 0.006; B‐cell: 0.417 (0.276–0.630), *p* < 0.001; NK‐cell: 1.202 (1.021–1.414), *p* = 0.027

*Note:* Cell‐count predictors were standardized; coefficients are adjusted odds ratios per 1 standard deviation increase.

Abbreviations: AUC, area under the receiver operating characteristic curve; CI, confidence interval; CRP, C‐reactive protein; NK, natural killer; VIF, variance inflation factor.

## 4 Discussion

This study clarifies how clinical flow cytometry should be interpreted alongside routine blood counts. We present three key findings. First, while the absolute T‐cell count strongly mirrors conventional ALC grading, this relationship is a mathematical inevitability driven by T‐cell dominance in peripheral blood; our T‐cell cutoffs therefore serve as an institutional calibration rather than a novel clinical classification. Second, simply replacing the ALC grade with a T‐cell grade does not improve the discrimination of hs‐CRP‐defined inflammation. Third, absolute B‐cell count provides the most consistent subset‐level signal, although the incremental discrimination was modest. Together, these results demonstrate that the clinical value of flow cytometry lies in capturing unique subset shifts, such as B‐cell depletion, rather than duplicating total lymphocyte information.

This distinction is not merely semantic. A high AUC for T‐cell count identifying ALC‐defined lymphopenia does not mean that T‐cell count has independently discovered a new clinical construct. Rather, it shows that a large component of the total lymphocyte pool can reproduce the behavior of the total count. The proposed T‐cell thresholds may still be useful for harmonizing flow cytometry reports with familiar CTCAE categories, especially in datasets where subset counts are emphasized, but their evidentiary status is closer to calibration than to independent validation.

Substituting a T‐cell grade for conventional ALC grade did not materially improve cross‐sectional discrimination of CRP‐defined inflammation. A full‐cohort sensitivity analysis that treated unmeasured CRP values as negative yielded uniformly lower discrimination, consistent with outcome misclassification under a clinically plausible missing‐not‐at‐random mechanism: CRP is commonly ordered when infection or inflammation is suspected.

The complete‐case analysis does not fully eliminate bias, because CRP measurement was not randomized. Clinicians may have ordered CRP because of fever, suspected infection, inflammatory disease activity, postoperative status, or other clinical concerns. Thus, the CRP‐measured subgroup is likely enriched for patients with a higher pretest probability of inflammation. Nevertheless, restricting the primary outcome analysis to patients with an observed CRP value avoids the more severe problem of assigning a negative outcome to patients whose outcome was never measured. Alternative missing‐data approaches, including multiple imputation, would require stronger assumptions and richer clinical covariates than were available here [23]. Consequently, these results apply specifically to hospitalized patients in whom clinicians considered CRP measurement clinically indicated and should not be generalized to the overall unselected hospitalized population.

In addition, B‐cell count appears to carry the most consistent incremental subset signal, although its discrimination remains modest and not definitively superior to all other markers without external validation. The biologic plausibility of B‐cell depletion and infection susceptibility is strong, but the present cross‐sectional dataset cannot establish causality, timing, or medication‐mediated mechanisms [24].

The B‐cell finding should therefore be read as hypothesis‐generating. It is compatible with known clinical contexts in which impaired humoral immunity accompanies infection susceptibility, such as anti‐CD20 therapy, hypogammaglobulinemia, hematologic malignancy, or combined immunosuppression [24]. However, the dataset does not identify these conditions or medication exposures, and the outcome is CRP elevation rather than adjudicated infection. A future study with diagnoses, immunoglobulin levels, medication history, microbiologic results, and infection sites would be needed to determine whether B‐cell count adds clinically actionable information beyond routine variables.

The multivariable results require caution. A model containing T, CD4, CD8, B, and NK counts is structurally collinear because CD4 and CD8 are major components of T cells and the major subsets together approximate ALC. Collinearity can destabilize coefficients and complicate interpretation when correlated predictors are entered together [25]. The observed NK coefficient reversal is therefore more consistent with suppression or collinearity than with proven compensatory NK expansion. For inference, parsimonious models such as conventional grade plus B‐cell count or a nonredundant T + B + NK panel are more defensible.

This issue also illustrates why apparent model improvement must be interpreted cautiously. Adding more correlated predictors can increase apparent AUC in the derivation dataset while producing unstable coefficients and weaker performance under internal validation. In the present analysis, cross‐validated areas under the receiver operating characteristic curve remained modest even for the more complex models. The clinically relevant message is therefore not that a particular multi‐marker equation is ready for use, but that subset composition may contain limited complementary information that should be evaluated in better‐characterized cohorts.

Several limitations remain. This was a retrospective single‐center analysis of patients selected for clinical flow cytometry. CRP is nonspecific and was measured in only a subset of patients, with likely missing‐not‐at‐random ordering. The dataset lacks age, sex, diagnosis, comorbidities, immunosuppressive therapy, microbiology, infection site, temporal ordering, and outcomes. All cutoffs were derived internally, and all associations are unadjusted for clinical confounding. External validation and prospectively defined infection outcomes are needed before clinical implementation. In particular, the absence of information on active hematologic malignancy, autoimmune disease, anti‐CD20 therapy, systemic corticosteroids, chemotherapy, and other immunomodulatory treatments prevents separation of disease‐related from treatment‐related lymphocyte‐subset depletion and leaves substantial residual confounding.

Despite these limitations, the analysis clarifies an important practical point. Flow cytometry should not be valued merely as a more complicated substitute for total lymphocyte count. Its potential value lies in describing immune composition, especially when specific compartments such as B cells may be disproportionately depleted. A balanced interpretation is therefore to use T‐cell count as a bridge to conventional grading when needed, while avoiding claims that T‐cell‐based grading alone improves inflammation assessment. CRP remains inexpensive, rapid, and widely available, whereas lymphocyte‐subset analysis is more costly, less accessible, and operationally more demanding. Our findings therefore do not support ordering flow cytometry as a substitute for CRP. Its potential complementary role is limited to settings in which subset testing is already clinically indicated, such as suspected or established immunodeficiency, hematologic malignancy, transplantation, or monitoring of B‐cell‐depleting or other immunosuppressive therapy. In those settings, subset composition may contextualize immune vulnerability alongside ALC and CRP, but the modest discrimination observed here and the lack of adjudicated clinical outcomes preclude practice‐changing thresholds.

## 5 Conclusions

T‐cell absolute count is an excellent within‐dataset surrogate for conventional ALC‐based lymphopenia grade, but this largely reflects the mathematical relationship between a dominant lymphocyte subset and total lymphocyte count. For CRP‐defined inflammation, conventional grade, T‐cell grade, and individual subset counts provide only modest cross‐sectional discrimination. B‐cell count provides the most reproducible incremental signal in this dataset, while redundant multi‐subset models should be interpreted cautiously because of severe collinearity and lack of clinical confounder adjustment. Lymphocyte‐subset enumeration should not replace CRP testing; any value is complementary and most plausible when flow cytometry is already indicated for a specific immune‐monitoring question.

## Author Contributions

**Shicheng Li:** conceptualization, methodology, formal analysis, data curation, visualization, and writing – original draft. **Tingting Fu:** investigation, data curation, and writing – review and editing. **Yang Yang:** methodology, validation, and writing – review and editing. **Yuwei Pu:** investigation, resources, and writing – review and editing. **Wenying Yan:** conceptualization, supervision, project administration, and writing – review and editing. **Junjun Zhang:** conceptualization, supervision, project administration, funding acquisition, and writing – review and editing. All authors accepted responsibility for the entire content of this manuscript and approved its submission.

## Funding

This study was funded by the Suzhou Applied Basic Research Technology Innovation Project (SYW2024096); Scientific Research Program for Young Talents of China National Nuclear Corporation (Junjun Zhang); Open Project of the State Key Laboratory of Radiology and Radiation Protection of Soochow University (GZK1204048); Fundamental Research Funds for the Central Universities; JLU Fundamental Research Funds for the Suzhou Key Lab of Multi‐modal Data Fusion and Intelligent Healthcare (25SZZD02); and Suzhou Sports Bureau Sports Science Research Project (TY2025‐203).

## Disclosure

No previously published material is reproduced. Clinical Trial Registration: this retrospective observational study did not assign participants to an intervention.

## Ethics Statement

This study was conducted in accordance with the Declaration of Helsinki and was approved by the Ethics Committee of the Second Affiliated Hospital of Soochow University (approval no. JD‐HG‐2026‐044).

## Consent

The Ethics Committee waived the requirement for written informed consent because of the retrospective, anonymized design.

## Conflicts of Interest

The authors declare no conflicts of interest.

## Supporting information


**Table S1:** Same‐data performance of individual flow cytometric markers for identifying conventional lymphopenia thresholds.
**Figure S1:** Distribution of T, CD4+ T, CD8+ T, B, and natural killer absolute counts across conventional lymphocyte grades. Box plots show the median, interquartile range, and 5th–95th percentile whiskers on a log‐scaled y‐axis.
**Figure S2:** CRP ≥ 10 mg/L proportions among patients with measured CRP by conventional grade and T‐cell grade. Bars show observed proportions; whiskers show Wilson 95% confidence intervals; labels below bars indicate measured‐CRP denominators.

## Data Availability

The data underlying this article are available from the corresponding authors upon reasonable request, subject to institutional and ethical requirements.
