# Supplementary material for: Flow Cytometric Lymphocyte Subset Enumeration for Lymphopenia Grading and C‐Reactive Protein‐Defined Inflammation Discrimination
Source: J Clin Lab Anal. 2026 Oct 15;40(21):e70375. doi: 10.1002/jcla.70375 (PMC13673062; doi:10.1002/jcla.70375)
Supplement: Supplementary file 1 [file JCLA-40-e70375-s001.docx]

**Supplementary Material**

Flow cytometric lymphocyte subset enumeration for lymphopenia grading and C-reactive protein-defined inflammation discrimination

Shicheng Li, Tingting Fu, Yang Yang, Yuwei Pu, Wenying Yan, and Junjun Zhang

**Supplementary Table S1: Same-data performance of individual flow cytometric markers for identifying conventional lymphopenia thresholds.**

| **Marker** | **Threshold** | **AUC** | **95% CI** | **Youden cutoff** | **Sensitivity** | **Specificity** |
| --- | --- | --- | --- | --- | --- | --- |
| T lymphocytes | Any lymphopenia (Grade ≥1) | 0.933 | 0.923-0.941 | 700.0 | 0.876 | 0.851 |
| T lymphocytes | Moderate-severe (Grade ≥2) | 0.942 | 0.934-0.951 | 573.0 | 0.934 | 0.817 |
| T lymphocytes | Severe (Grade ≥3) | 0.962 | 0.945-0.976 | 356.0 | 0.896 | 0.930 |
| CD4+ T cells | Any lymphopenia (Grade ≥1) | 0.902 | 0.891-0.912 | 365.0 | 0.818 | 0.826 |
| CD4+ T cells | Moderate-severe (Grade ≥2) | 0.910 | 0.900-0.920 | 296.0 | 0.859 | 0.807 |
| CD4+ T cells | Severe (Grade ≥3) | 0.943 | 0.930-0.955 | 218.0 | 0.926 | 0.846 |
| CD8+ T cells | Any lymphopenia (Grade ≥1) | 0.843 | 0.828-0.856 | 312.0 | 0.799 | 0.729 |
| CD8+ T cells | Moderate-severe (Grade ≥2) | 0.863 | 0.849-0.876 | 263.0 | 0.850 | 0.727 |
| CD8+ T cells | Severe (Grade ≥3) | 0.907 | 0.884-0.927 | 178.0 | 0.832 | 0.834 |
| B lymphocytes | Any lymphopenia (Grade ≥1) | 0.788 | 0.770-0.803 | 72.0 | 0.694 | 0.747 |
| B lymphocytes | Moderate-severe (Grade ≥2) | 0.808 | 0.790-0.825 | 56.0 | 0.756 | 0.723 |
| B lymphocytes | Severe (Grade ≥3) | 0.871 | 0.846-0.893 | 26.0 | 0.802 | 0.840 |
| NK cells | Any lymphopenia (Grade ≥1) | 0.804 | 0.788-0.819 | 237.0 | 0.826 | 0.625 |
| NK cells | Moderate-severe (Grade ≥2) | 0.842 | 0.827-0.856 | 156.0 | 0.771 | 0.752 |
| NK cells | Severe (Grade ≥3) | 0.894 | 0.875-0.912 | 117.0 | 0.871 | 0.777 |

AUC, area under the receiver operating characteristic curve; CI, confidence interval; NK, natural killer.

**Supplementary Figure S1: Distribution of T, CD4+ T, CD8+ T, B, and natural killer absolute counts across conventional lymphocyte grades. Box plots show the median, interquartile range, and 5th-95th percentile whiskers on a log-scaled y-axis.**

**Supplementary Figure S2: CRP ≥10 mg/L proportions among patients with measured CRP by conventional grade and T-cell grade. Bars show observed proportions; whiskers show Wilson 95% confidence intervals; labels below bars indicate measured-CRP denominators.**
